# Predictive and diagnostic biomarkers for gestational diabetes and its associated metabolic and cardiovascular diseases

**DOI:** 10.1186/s12933-019-0935-9

**Published:** 2019-10-30

**Authors:** A. Lorenzo-Almorós, T. Hang, C. Peiró, L. Soriano-Guillén, J. Egido, J. Tuñón, Ó. Lorenzo

**Affiliations:** 1 Renal, Vascular and Diabetes Laboratory, Instituto de Investigaciones Sanitarias-Fundación Jiménez Díaz, Universidad Autónoma de Madrid, Av. Reyes Católicos 2, 28040 Madrid, Spain; 2 Department of Pharmacology, School of Medicine, Universidad Autónoma de Madrid, Madrid, Spain; 3 Department of Paediatrics, IIS-Fundación Jiménez Díaz, UAM, Madrid, Spain; 4 Department of Cardiology, Fundación Jiménez Díaz, Madrid, Spain; 5 Spanish Biomedical Research Centre in Diabetes and Associated Metabolic Disorders (CIBERDEM) Network, Madrid, Spain

**Keywords:** Gestational diabetes, Predictive biomarkers, Diagnostic biomarkers, Metabolic disease, Cardiovascular disease

## Abstract

Gestational diabetes mellitus (GDM) is defined as the presence of high blood glucose levels with the onset, or detected for the first time during pregnancy, as a result of increased insulin resistance. GDM may be induced by dysregulation of pancreatic β-cell function and/or by alteration of secreted gestational hormones and peptides related with glucose homeostasis. It may affect one out of five pregnancies, leading to perinatal morbidity and adverse neonatal outcomes, and high risk of chronic metabolic and cardiovascular injuries in both mother and offspring. Currently, GDM diagnosis is based on evaluation of glucose homeostasis at late stages of pregnancy, but increased age and body-weight, and familiar or previous occurrence of GDM, may conditionate this criteria. In addition, an earlier and more specific detection of GDM with associated metabolic and cardiovascular risk could improve GDM development and outcomes. In this sense, 1st–2nd trimester-released biomarkers found in maternal plasma including adipose tissue-derived factors such as adiponectin, visfatin, omentin-1, fatty acid-binding protein-4 and retinol binding-protein-4 have shown correlations with GDM development. Moreover, placenta-related factors such as sex hormone-binding globulin, afamin, fetuin-A, fibroblast growth factors-21/23, ficolin-3 and follistatin, or specific micro-RNAs may participate in GDM progression and be useful for its recognition. Finally, urine-excreted metabolites such as those related with serotonin system, non-polar amino-acids and ketone bodies, may complete a predictive or early-diagnostic panel of biomarkers for GDM.

## Background

During pregnancy, women must adapt her body systems to support nutrient and oxygen supply for the growth of the fetus and subsequent lactation [1]. Inappropriate adaptation of maternal physiology may lead to complications of pregnancy, such as gestational diabetes mellitus (GDM). The American Diabetes Association (ADA) has categorized GDM as an independent type of diabetes, caused and developed by different mechanisms, and requiring specific diagnosis and treatment approaches [2]. GDM may originate from specific gene mutations and/or dysregulation of placental hormones and β-cell injury, and can be favoured by advanced age, gynaecological alterations and diabesogenic factors. GDM is usually developed after the 2nd trimester of pregnancy, between the 24th and the 28th week of gestation [1, 3], and it can trigger serious and long-term consequences for fetal and maternal health, in particular, those on metabolism and cardiovascular physiology [4]. According to the International Association of Diabetes and Pregnancy Study Groups (IADPSG), GDM may complicate 15–20% pregnancies, and has being augmented in the last 20 years in all ethnic groups as much as 27% [5]. However, the exact prevalence of GDM remains unknown, possibly due to the different diagnostic criteria.

The original classification of GDM, dated from 1949, was based on age at onset of GDM and on duration of associated metabolic and cardiovascular complications, ranging from “type A” (more favourable) to “type F” (more deleterious) [6]. Recently, new principles including glucose homeostasis, body weight and family history of GDM, have been considered for clinical practice. Also, a specific distinction has been made between pre-gestational diabetes and GDM, since the prevalence of diabetes along with overweight and obesity have been increasing worldwide at younger ages [7]. Unfortunately, current criteria for GDM diagnosis, based on glucose homeostasis, cannot anticipate or detect all GDM cases neither distinguish those women under cardiovascular risk [8]. The knowledge of new biomarkers for premature detection of GDM with/without associated cardiovascular risk could advance the appropriated management of these patients. In particular, alteration of those biomarkers would classify GDM subjects to initiate early adjustments of specific detrimental factors and tissue responses, in both mother and fetus.

## Maternal adaptations of metabolism and cardiovascular system during pregnancy

Specific metabolic and cardiovascular changes occur in women to acclimate to the pregnant state. Some modifications appear very early in pregnancy, even before the formation of a functional placenta [9]. The maternal pancreatic β-cell mass expands due to both hyperplasia and hypertrophy of islets, enhancing insulin secretion [10]. Thus, maternal insulin sensitivity is frequently increased, subsequently with lipogenesis and lipid storage at the adipose tissue. Also, alterations in cardiac size, morphology and function must respond to hormonal and metabolic demands. In this sense, the stimulation of systemic vasodilation, blood perfusion and cardiac output increases blood volume to handle with the oxygen and nutrient request [11]. However, later, by the 2nd–3rd trimester of pregnancy and coinciding with the fast growth of fetus, a decrease in insulin sensitivity and an induction of lipolysis and hepatic gluconeogenesis is observed [12]. Insulin receptors and signaling are also ameliorated, and β-cells react by producing more insulin to maintain an euglycemic state [13, 14]. Also, heart increases rate, wall thicknesses and contractility, stimulating ventricular compliance [15]. Importantly, evidence in human and mostly in animal models have confirmed a placental control of metabolism and cardiovascular homeostasis.

### Placental regulation of metabolism and cardiovascular system

From the 6th week of pregnancy, placenta releases a variety of molecules with physiological effects on metabolism and cardiovascular system for both mother and fetus [16]. Among placental factors, progesterone and oestrogen are key steroid hormones for controlling insulin sensitivity [17]. Both steroids prompt pancreatic hypertrophy, though progesterone reduces insulin-stimulated glucose uptake and oestrogen stimulates systemic insulin sensitivity. They also exert opposite effects on food intake and vascular physiology. Progesterone stimulates appetite and fat deposition, as well as neuropeptide-Y expression for vasoconstriction, whereas oestrogen promotes leptin-dependent satiety and vasodilatation [18]. Moreover, progesterone decreases cardiomyocyte apoptosis, and triggers metabolic shift from carbohydrate to lipid, as a main energetic substrate for the myocardium [19]. Other placental factors can also modulate metabolic and cardiovascular function during pregnancy. Leptin reduces food intake during gestation [20], and neuroactive hormones as melatonin and serotonin, improve glucose tolerance and insulin sensitivity [21]. Also, oxytocin reduces glucose and insulin intolerance, food intake and adiposity, and lowers blood pressure and cardiac oxidation/inflammation [22]. Furthermore, a fine metabolic and cardiovascular control will depend on release of the prolactin and growth hormone (PRL-GH) family. PRL induces β-cell proliferation and insulin secretion, while GH promotes the cardiac metabolic shift to lipid and reduces insulin signalling [23]. PRL triggers food intake through leptin inhibition, but GH decreases appetite by attenuation of ghrelin and neuropeptide-Y expression [24, 25]. Finally, activin-A and relaxin are also discharged to enhance glucose tolerance and vascular function [26, 27]. Thus, during pregnancy, maternal adaptations for metabolic and cardiovascular needs should be finely regulated by placental factors. The inappropriate and/or unbalanced delivery or action of these molecules might increase the risk of GDM and associated cardiovascular pathologies [16].

## GDM aetiology and associated risk factors

Given the prevalence of GDM along family members, a genetic predisposition has been suggested [28]. Some of the genetic variants for GDM coincide with those of type-II diabetes (T2DM) [29]. Mutations in insulin, insulin receptor, insulin-like growth factor-2, glucokinase, PRL-GH family, hepatocyte nuclear factor-4A, plasminogen activator inhibitor 1 (PAI-1) and melatonin receptor 1B, among others, have been recognized [30, 31]. Moreover, Chinese, Southeast Asian, Middle Eastern or Indian backgrounds were linked with higher prevalence of GDM [32]. However, the aetiology of GDM has been traditionally connected to a dysregulation of placental hormones favouring the discharge or effect of those that interfere with insulin sensitivity [14]. In fact, alteration in progesterone and oestrogen delivery was correlated with GDM development [33]. Also, leptin was up-regulated in GDM women [34] and mutant female mice in leptin receptor lead to spontaneous development of GDM during pregnancy [35]. Furthermore, other studies have concluded that GDM may be mainly originated from β-cell injury. The β-cell number can decrease 41% in GDM mothers [36], potentially due to delivery of “toxic” adipokines [37]. In this sense, high levels of IFN-γ and TNF-α from CD4^+^-Th1 cells damaged pancreatic islets by controlling the activation of transcription factors (i.e., FOXD3, FOXM1, HNF4α) and related proliferative and survival genes [38]. Also, dietary restriction of tryptophan reduced serotonin synthesis and β-cell expansion, leading to glucose intolerance and increased GDM risk [39].

In addition to genetic mutations, unbalanced hormone secretion and/or β-cell injury, some other potential risk factors have been suggested for GDM. In particular, the presence of polycystic ovarian syndrome and a clinical history of previous GDM or previous macrosomia in the new-born [28]. Moreover, the National Institute for Health and Clinical Excellence (NICE) and ADA concluded that increased age, and mainly body weight, correlated with GDM incidence [28]. Pregnant women under 20 years-old did not present GDM, whilst 33.3% of them showed GDM at 20–29 years-old, and 58.3% at 30–39 years-old [34]. Importantly, obesity can trigger GDM development. Although body mass index (BMI) is not appropriately descriptive for obesity during pregnancy, this anthropomorphic parameter was linked to GDM occurrence [40]. Adipose tissue and placenta can produce a similar pattern of cytokines, which explains the fact that obese women are at higher risk of GDM [41]. Thus, an excessive body weight is frequently present in GDM women, worsening maternal (and fetal) alterations in metabolism and cardiovascular system [42, 43]. Indeed, maternal obesity and GDM may be associated with a state of chronic, low-grade inflammation by which offspring are programmed to develop adult disorders [44]. In this line, intakes of fat and sweet diets before gestation were also associated with elevated risk of GDM, whereas meals based on fruits, vegetables and fish provoked opposite trends [45]. The low ingestion of polyunsaturated fat [46], ascorbic acid [47] and vitamin D [48] were related to GDM, but addition of fibre to diets reduced its prevalence [49]. Also, overproduction of ketone bodies [i.e., α-hydroxybutyrate (AHBA)], typical in obesity, paralleled the impairment of insulin secretion during gestation and GDM [50]. In addition, maternal obesity can promote by itself birth defects in offspring. A recent metanalysis collecting data from 1980 has provided robust evidence of a positive association between maternal BMI and the risk for fetal congenital heart defects [51]. Altogether, predisposition to GDM may be favoured by age and diabesogenic factors, and influenced by previous gynaecological alterations of the subject.

## Maternal pathologies associated to GDM development

A systemic low-grade inflammation is physiologically prompted during pregnancy by humoral immunity in order to maintain a safe environment and to avoid fetal rejection [52]. However, by transcriptomics, Radaelly’s laboratory found in placenta a highly expressed pro-inflammatory pattern mainly of endothelial factors, reflecting chronic inflammation with signs of major vascular dysfunction [53]. Among these factors, receptors for interleukin (IL)-8, IL-1 and leptin, together with pentraxin-related gene (PTX-3) were upregulated. Other authors demonstrated high levels of pro-inflammatory serum high sensitive C-reactive protein (hs-CRP) [54], E-selectin, osteoprotegerin, adhesion molecules (VCAM-1, ICAM-1), symmetric dimethylarginine (SDMA) and a disintegrin and metalloproteinase (ADAM) [55]. Similarly, a pro-inflammatory pattern of upregulated adipokines (i.e., IL-6 and hs-CRP) and diminished anti-inflammatory adiponectin was also observed in adipose tissue from GDM women [56]. Remarkably, this pro-inflammatory milieu, together with the dysregulated secretion of placental factors and/or β-cell injury, could trigger metabolic and cardiovascular diseases in GDM women and offspring [57].

### Metabolic and cardiovascular disorders in GDM women

The risk of T2DM in women after GDM is elevated in the first 5 years [58], raising up to 50% risk after 10 years [59], and 70% after 28 years [60]. Other study established a ten times more likely to develop T2DM within 10 years compared to normal pregnancies [61]. Also, GDM has been related with the development of post-parturition metabolic syndrome, mainly in obese women [62]. Fasting glucose, insulin resistance and β-cell dysfunction remained after pregnancy [63]. Levels of E-selectin and ICAM-1, fibrinogen, IL-6, tissue inhibitor of metalloproteinase-1 (TIMP-1) and PAI-1 [60, 64, 65], but not adiponectin [66], were prominent in women with previous GDM. Also, subclinical inflammation associated with elevated levels of TIMP-1 were observed in women 4 years after GDM [44]. However, it is not clear whether GDM may be an independent cause of these anomalies or they can be a consequence of related comorbidities such as atherosclerosis, hypertension or obesity [67]. In this sense, the development of postpartum T2DM and metabolic syndrome was independently correlated with endothelial dysfunction and increased carotid intima-media thickness in GDM women [65].

On the other hand, maternal cardiovascular adaptations (i.e., increased heart rate, ventricular walls and vasodilatation) return after delivery in normal pregnancies. Cardiac output also decreases within the first hour postpartum and reaches baseline levels after 2 weeks [68]. However, GDM gestations have been linked with subclinical alterations in cardiac structure (i.e., increased thicknesses of left ventricular wall and intraventricular septum) and diastolic dysfunction [69]. Also, it was related to preeclampsia and vasculopathies, specifically, arterial stiffness, endothelial dysfunction and atherosclerosis [44, 70, 71]. More worrying, GDM has been positively linked with a 66% increase of long-term cardiovascular injuries [72]. An elevated rate of hospitalizations due to cardiovascular failures, and independently of high BMI, was detected in GDM women after parturition [43]. The long-term US CARDIA (Coronary Artery Risk Development in Young Adults) registry demonstrated an increase of left ventricular mass, and abnormalities in left ventricular relaxation and systolic dysfunction in 609 women, 20 years after GDM [73]. Also, left ventricular hypertrophy and diastolic dysfunction remained 8 weeks after delivery [74], and elevation of the triglyceride/HDL-lipoprotein ratio was associated with previous GDM in 300 women after 5 years of childbirth [75]. In addition, GDM was associated with a 56% higher risk of upcoming cardiovascular events, and a 2.3-fold increased risk of cardiovascular incidents in the first decade postpartum, independently of progression to T2DM [76]. Coronary artery disease and stroke were greater in 332 women with previous GDM, independently of T2DM, metabolic syndrome or obesity incidence [77]. Increased rates of myocardial infarction and angina pectoris were observed 7 years after delivery, but, however, were stimulated by obesity, advanced age and hypertension [43].

## Fetal and child comorbidities associated with GDM

Since GDM develops from the 2nd–3rd trimester of pregnancy, GDM pregnancies have not been associated with congenital malformations as pre-gestational diabetes do [78]. However, GDM represents high risk for perinatal morbidity and adverse neonatal outcomes compared to normal pregnancies. The excess of plasma glucose and lipids in GDM mothers was linked to cardiac hypertrophy [79] and dysfunction [80] in fetus. Hyperinsulinemia promoted insulin resistance, which also stimulated cardiac hypertrophy [81]. Also, the increased glucose, amino acids, and fatty acids assimilation observed in GDM placenta, stimulated endogenous fetal production of insulin and insulin-like growth factor-1 (IGF-1), which induced macrosomia [82]. Thus, the risk of stillbirths after GDM is four times higher than in normal pregnancies [83]. Later, neonates from GDM are at increased risk of hypoglycemia due to the high dependence on maternal hyperglycemia. Children and adolescents can reach higher BMI, glucose intolerance and hypertension, independently of macrosomia at birth [84]. Also, they exhibited impairment of diastolic function as a prolonged deceleration time associated with early left ventricular diastolic filling [81]. Furthermore, females are more likely to experience GDM in their own pregnancies, contributing to a vicious intergenerational cycle of this pathology [30].

## GDM treatment

Early intervention for GDM could be crucial to prevent subsequent damage in both mother and fetus [85]. Women with GDM are recommended to initiate a change of lifestyle, as well as pharmaceutical treatment, if needed [86]. For non-obese women with GDM, diets containing 30–35 kcal per kg of body weight, with 33–40% calories from carbohydrates, are advised [49]. Also, practical exercise before and during pregnancy can preserve glucose homeostasis and improve GDM pathology [87]. In particular, moderate exercise (30 min—5 times/week) has demonstrated attenuation of insulin resistance, GDM and fetal macrosomia in obese and non-obese women [88]. More intense activities (> 60 min) could, however, provoke hypoglycaemia [89].

Thereafter, if glycemic target is not achieved after 1–2 weeks of lifestyle changes, the American College of Obstetricians and Gynaecologists (ACOG) and NICE guidelines recommend pharmacotherapy [90]. In fact, maternal hyperglycemia and advanced age or BMI by themselves are already indicators of medical requirement [91]. Regarding glucose control, rapid-acting insulin analogues, long-acting insulin or even premixed preparations can be useful for GDM. Unfortunately, hypoglycemia is frequent in some subjects, suggesting the need of alternative administrations, such as those of high doses of intermittent insulin injections. Also, sulfonylureas (i.e., glyburide) may produce similar effects than insulin, but cannot mitigate neonatal hypoglycaemia and macrosomia [92]. Interestingly, metformin reduces hyperglycemia and weight gain more intensively than insulin, though metformin does not decrease neonatal hypoglycaemia or macrosomia [93]. Thus, recent data have suggested a lifestyle modification followed by glyburide or metformin, when fasting glucose is between 95 and 114 mg/dL, or a combination of both drugs, when glucose is 115–125 mg/dL. GDM over 126 mg/dL, should be treated with insulin [94]. New strategies addressing insulin homeostasis as well as adiposity, while protecting cardiovascular system, could be of special interest [95–97]. However, pharmacological treatments might negatively affect either mother and offspring with variable degree depending on age and background, pregnancy stage and the presence of comorbidities [98]. An adequate prediction or early diagnosis of GDM by specific, safe and minimally-invasive approaches could reduce short and long-term abnormalities in both mother and offspring.

## GDM diagnosis

Currently, there is not a standardised methodology for GDM identification. Universal or selective screening, different glucose tests and diverse glycemia cut-off values, are being recognized. These criteria also vary among countries and between obstetric and diabetes organizations [8]. Some international (IADPSG [99]) and national (NICE [100]; the German Association for Gynaecology and Obstetrics, DGGG [101]; the Journal of Obstetrics and Gynaecology Canada, JOGC [102]; and the National Institutes of Health, NIH [103]) associations suggest in their guidelines a screening for GDM prediction at the first prenatal visit with gynaecologist (Table 1). After quantifying glucose homeostasis based on different parameters [fasting glucose, random glucose or oral glucose tolerance tests (OGTT) following glucose overload], GDM can be predicted if specific cut-offs are reached, and therapeutic programs are recommended. Otherwise, women will be evaluated again at the third trimester. In contrast, since high levels of glycosylated haemoglobin (HbA1c) unveiled a (modest) correlation with GDM only between the 24th and the 28th week of pregnancy [104], other associations (ACOG [105]; ADA [106]; and the International Federation of Gynaecology and Obstetrics, FIGO [107]) directly advise the screening of GDM at this stage (Table 2). In the one-step strategy, GDM is identified by quantification of glucose homeostasis at the fasting state and after 1–2 h glucose overload. In the two-step routine, GDM is diagnosed when detected hyperglycemia by a glucose challenge test (GCT) is confirmed by another 1–3 h-glucose surplus.

**Table 1:** Current criteria for GDM prediction

Association	Screening type	Screening approach (first pre-natal visit)	Cut-offs for GDM prediction
IADPSG	Universal	Fasting plasma glucose test	Fasting glycemia ≥ 92 mg/dL (5.1 mM) predict GDM^d^
DGGG	High risk women^a^	Random plasma glucose test	Glucose ≥ 200 mg/dL (11.1 mM) proceed with fasting plasma glucose test Glucose 140–199 mg/dL (7.8–11.0 mM) proceed with fasting plasma glucose test or OGTT
NICE	Women with previous GDM	One-step strategy (2 h OGTT for 75 g glucose overload)	Fasting glycemia ≥ 100.8 mg/dL (5.6 mM) Glycemia 2 h after overload ≥ 140.4 mg/dL (7.8 mM)
NIH	High risk women^b^	Two-steps strategy (1 h GCT for 50 g glucose overload + 3 h 100 g glucose overload)	Step 1: If glycemia ≥ 130 mg/dL (7.2 mM), proceed with Step 2^e^: Fasting glycemia ≥ 95 mg/dL (5.3 mM) Glycemia 1 h after overload ≥ 180 mg/dL (10.0 mM) Glycemia 2 h after overload ≥ 155 mg/dL (8.6 mM) Glycemia 3 h after overload ≥ 140 mg/dL (7.8 mM)
JOGC	High risk women^c^	Two-steps strategy (1 h GCT for 50 g glucose overload + 2 h OGTT for 75 g glucose overload)	Step 1: If glycemia ≥ 200 mg/dL (11.1 mM), GDM is diagnosed If glycemia ≥ 140–200 mg/dL (7.8–11.1 mM), proceed with Step 2: Fasting glycemia ≥ 95 mg/dL (5.3 mM) Glycemia 1 h after overload ≥ 190 mg/dL (10.6 mM) Glycemia 2 h after overload ≥ 162 mg/dL (9.0 mM)

After universal or selective screening of pregnant women at the first pre-natal visit, diabetic and obstetrician associations preferentially recommend specific strategies for GDM prediction. Basing on glucose homeostasis, different approaches can be followed. The estimation of GDM is made when any or two (in the 3 h OGTT) cut-offs are met

*OGTT* oral glucose tolerance test, *GCT* glucose challenge test

^a^Age ≥ 45 years-old, pre-gestational BMI ≥ 30 kg/m^2^, familiar or previous GDM, DM or macrosomia, Asian and Latin American ethnicities, arterial hypertension, dyslipidemia, polycystic ovary syndrome, and history of coronary or cerebral vascular disease

^b^Similar to DGGG, but including age ≥ 25 years-old, persistent glucosuria, history of spontaneous abortions and unexplained stillbirths

^c^Similar to DGGG, but including age ≥ 35 years-old, and aboriginal and African ethnicities

^d^If fasting glycemia ≥ 126 mg/dL (7.0 mmol/L), DM should be considered

^e^Following Carpenter/Coustan conversion method

**Table 2:** Current criteria for GDM diagnosis

Association	Screening type	Screening approach (24th–28th week)	Cut-offs for GDM diagnosis
ADA	High risk women^a^	One-step strategy (2 h OGTT for 75 g glucose overload)	Fasting glycemia: 92–125 mg/dL (5.1–6.9 mM)^b^ Glycemia 1 h after overload ≥ 180 mg/dL (10.0 mM) Glycemia 2 h after overload: 153–199 mg/dL (8.5–11.0 mM)^c^
IADPSG	Universal		
FIGO	Universal		
DGGG	Universal		
NICE	Universal	One-step strategy (2 h OGTT for 75 g glucose overload)	Fasting glycemia ≥ 100.8 mg/dL (5.6 mM) Glycemia 2h after overload ≥ 140.4 mg/dL (7.8 mM)
ACOG	Universal	Two-steps strategy (1 h GCT for 50 g glucose overload + 3 h OGTT for 100 g glucose overload)	Step 1: If glycemia ≥ 130 mg/dL (7.8 mM), proceed with Step 2^d^: Fasting glycemia ≥ 95 mg/dL (5.3 mM) Glycemia 1h after overload ≥ 180 mg/dL (10.0 mM) Glycemia 2 h after overload ≥ 155 mg/dL (8.6 mM) Glycemia 3 h after overload ≥ 140 mg/dL (7.8 mM)
NIH	Universal		
JOGC	Universal	Two-steps strategy (1 h GCT for 50 g glucose overload + 2 h OGTT for 75 g glucose overload)	Step 1: If glycemia ≥ 200 mg/dL (11.1 mM), GDM is diagnosed If glycemia ≥ 140–200 mg/dL (7.8–11.1 mM), proceed with Step 2: Fasting glycemia ≥ 95 mg/dL (5.3 mM) Glycemia 1 h after overload ≥ 190 mg/dL (10.6 mM) Glycemia 2 h after overload ≥ 162 mg/dL (9.0 mM)

After screening of pregnant women at the third trimester, the associations’ guidelines preferentially suggest specific approaches for GDM detection. One-step or two-steps schemes can be followed. The diagnosis of GDM is made when any or two (in the 3 h OGTT) cut-offs are met

^a^Age ≥ 25 years-old, BMI > 25 kg/m^2^, Asian and Latin American ethnicities, previous history of abnormal glucose tolerance or adverse obstetrics outcomes, and familiar history of DM

^b^If fasting glycemia ≥ 126 mg/dL (7.0 mM), T2DM should be considered

^c^If glycemia 2 h after overload ≥ 200 mg/dL (11.1 mM), T2DM should be contemplated

^d^Following Carpenter/Coustan conversion method

Nevertheless, these criteria based on glucose homeostasis might not anticipate or detect all GDM cases neither distinguish those women under cardiovascular risk [8]. In this sense, alternative biomarkers for assessing glycemic control have been proposed in GDM diagnosis. The 1-deoxy form of glucose, known as 1,5-anhydroglucitol, is a naturally occurring dietary polyol. Serum 1,5-anhydroglucitol competes with very high levels of glucose for reabsorption into the kidney, and thus, lower 1,5-anhydroglucitol levels can reflect hyperglycemia and glycosuria [108]. Interestingly, first trimester measurement of 1,5-anhydroglucitol was a valid biomarker for later onset of GDM [109]. In addition, the diagnostic window-period at the third trimester might be late to avoid chronic abnormalities in metabolism and cardiovascular system, in both mother and fetus. Therefore, new specific predictive and diagnostic tools should be evaluated for these patients.

## Prospective diagnostic and predictive markers for GDM with associated cardiovascular risk

**Fig. 1:**
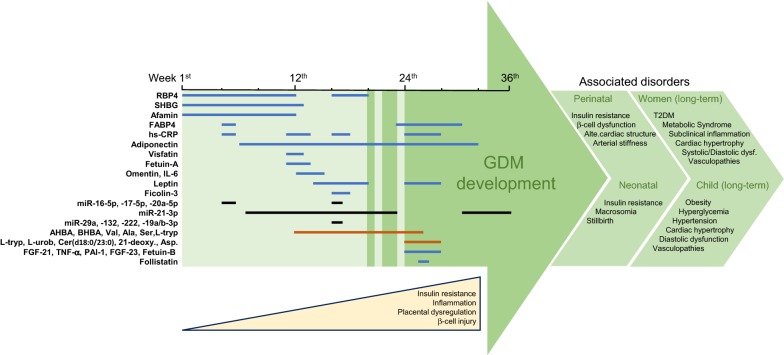
Predictive and diagnostic biomarkers for GDM pregnancies. GDM usually develops from the 2nd trimester of pregnancy in correlation with increased inflammation, insulin resistance, placental dysregulation and/or β-cell disruption, and can be detected at the 24th–28th week by evaluation of glucose homeostasis. However, some specific protein (blue lines), miRs (black lines) and metabolites (red lines) are released into the blood and/or urine from early stages of (complicated) pregnancies and could serve as biomarkers for GDM. In particular, RBP4, SHBG, afamin, FABP4, hs-PCR, adiponectin and several miRs (miR-16-5p, -17-5p, -20a-5p) could be tested at the beginning of pregnancies, mainly in women with risk factors (obesity, advanced aged, previous GDM). In addition, visfatin, fetuin-A, omentin, leptin, ficolin-3 and specific metabolites (i.e., AHBA, L-Tryp) may be useful for the mid-stage of gestation, and FGF-21, PAI-1, fetuin-B and follistatin, and other metabolites [Ceramide (d18:0/23:0), aspartame] could help GDM screening at the 3rd trimester. Then, early interventions on metabolic and cardiovascular abnormalities could attenuate associated post-parturition (perinatal, neonatal and chronic) disorders in women and offspring

During GDM, the dysfunctional adipose tissue and placenta may secrete specific, stable and easy-to-quantify factors, which may participate in inflammation, insulin resistance and cardiovascular injuries. These soluble biomarkers could be found in maternal circulation or urine and might be used for GDM prediction and/or detection, and provide information about the risk of associated metabolic and cardiovascular diseases (Fig. 1).

i. Adipose tissue-derived factors

Adipose tissue is an endocrine organ capable of secreting factors (adipokines) with paracrine functions. Some of these molecules could be implicated in promotion and progression of DM and cardiovascular injuries. In particular, leptin is a proinflammatory adipokine involved in immune responses that affects glucose metabolism by antagonizing appetite and insulin action. It also stimulates oxidative stress, arterial stiffness, and atherogenesis [110]. Interestingly, leptin levels have been revealed significantly higher from the 2nd half of pregnancy in normal and overweight women with later GDM diagnosis [111–113] (Table 3A). In contrast, adiponectin, an adipokine with anti-inflammatory, anti-atherosclerotic and insulin-sensitizing proprieties showed constantly lower levels along the 1st–3rd trimester of GDM gestations [113–115]. In fact, hypoadiponectinemia increased by 4.6 times the risk of developing GDM [116], and it was inversely correlated with BMI, insulin resistance and leptin [117]. Thus, the ratio of plasma adiponectin/leptin (< 0.33) has been also suggested as predictor of GDM as early as the 6th–14th week of pregnancy [118]. Nevertheless, further investigation analysing the value of the high-molecular weight oligomeric adiponectin could improve these estimations [119].

**Table 3:** Candidates biomarkers for GDM prediction

Panel A				
Protein biomarker	Main proposed origin	Week of pregnancy	Change in GDM	Metabolic- and cardiovascular-related properties
RBP4	Liver, adipose, breast	1st–12th/16–20th	Higher	Pro-inflammatory Glut4 down-regulation and insulin resistance Endothelial dysfunction
SHBG	Liver, placenta	1st–13th	Lower	Polycystic ovary syndrome Insulin resistance
Afamin	Liver, placenta	1st–12th	Higher	Insulin resistance Metabolic syndrome
FABP4	Adipose, placenta	4–6th/23rd–30th	Higher	Fatty acid uptake, transport, and metabolism
hs-CRP	Liver, pancreas, adipose	4–6th/11–14th 16–18th/24–28th	Higher	Pro-inflammatory of acute response
Adiponectin	Adipose, breast	6th–32nd	Lower	Anti-inflammatory and anti-atherogenesis Insulin-sensitizer
Visfatin	Adipose, placenta	11–13th	Higher	Pro-inflammatory and chemotactic Endothelial dysfunction Acute myocardial infraction
Fetuin-A	Liver, placenta, fetal tissues	11–14th	Lower	Pro-inflammatory Regulation of the insulin receptor Vessel calcification
Omentin-1	Adipose, placenta	12–15th	Lower	Anti-inflammatory Vasodilatation and endothelial function
IL-6	Adipose, lung	12–15th	Higher	Pro-inflammatory Atherogenesis and DM
Leptin	Adipose, breast	14–20th/24–28th	Higher	Reduction on insulin action and appetite Pro-oxidant and pro-inflammatory Arterial stiffness
Ficolin-3	Liver, placenta	16th–18th	Lower	Insulin resistance T2DM development

Panel B				
Genetic biomarker	Main proposed origin	Week of pregnancy	Change in GDM	Metabolic- and cardiovascular-related properties
miR-16-5p	Placenta	4–6th/16th	Higher	Pro-inflammatory Regulation of vascular endothelial growth
miR-17-5p	Placenta	4–6th/16th	Higher	Insulin resistance Regulation of angiogenesis Hypertension
miR-20a-5p	Placenta	4–6th/16th	Higher	Regulation of LDL receptor Modulation of aerobic cardiac capacity Coronary artery disease
miR-21-3p	Placenta	7th–23rd/30–36th	Higher	Pro-inflammatory Insulin resistance
miR-29a	Placenta	16th	Lower	Repression of insulin-signaling Regulation of Glut4 Control of fatty acid/glucose metabolism
miR-132	Placenta	16th	Lower	Insulin secretion Enhancement of glucose homeostasis
miR-222	Placenta	16th	Lower	Insulin resistance Downregulation of Glut4 Hypercholesterolemia
miR-19a/b-3p	Placenta	16th	Higher	Pro-inflammatory Insulin resistance Vascular injury

Some protein (A) and miRs (B) from diverse origins can be early detected in maternal plasma during gestation. Their modified levels have been correlated with later GDM development. Some of them can also provide information about potential metabolic and cardiovascular disorders (https://www.genecards.org/)

Moreover, proinflammatory adipokines that recruit and activate immune cell subsets in the white adipose tissue, could be quantified. Classical cytokines as hs-CRP and TNFα were higher in the serum from GDM women compared to healthy subjects during the 1st, 2nd and 3rd trimester of pregnancy [120–122] (Tables 3A and 4). PAI-1, a member of the superfamily of serpins that inhibit pro-coagulant plasminogen, was also significantly augmented at the 24–28th week [122], and was considered an early feature of the cardiometabolic biomarker profile of women with recent gestational dysglycemia [123]. Other potential adipokines for GDM diagnosis are visfatin, resistin and omentin. The former is an pro-inflammatory adipose mediator that promote endothelial dysfunction, atherosclerosis and acute myocardial infarction. It is also increased in patients with T2DM, metabolic syndrome or obesity [124]. Despite of its insulin-like properties by binding to the insulin receptor-1 and promotion of hypoglycaemic effects, visfatin can activate NFκB signalling and chemotaxis, contributing to the development of insulin resistance. Interestingly, visfatin was found increased at the late 1st trimester (Table 3A) [125], but differentially expressed at the 3rd trimester of GDM [126, 127]. Similarly, resistin, another small adipokine hormone related with high levels of LDL-c and pro-inflammatory molecules, was reduced or unchanged during GDM [128, 129]. However, omentin-1, an adipokine expressed in non-fat cells from adipose tissue (i.e., stromal vascular cells) and involved in vascular tone relaxation by production of endothelial nitric oxide and reduction of both hs-CRP and TNFα signalling [130], was decreased at the 2nd trimester of GDM in parallel to adiponectin, and in contrast to IL-6 [119] (Table 3A).

**Table 4:** Prospective biomarkers for GDM diagnosis

Protein biomarker	Main proposed origin	Week of pregnancy	Change in GDM	Metabolic- and cardiovascular-related properties
FGF-23	Adipose, liver	24–28th	Higher	Arterial stiffness Left ventricular hypertrophy
FGF-21	Liver, placenta	24–28th	Higher	Reduction of diabetes-associated vascular injury Stimulation of glucose uptake Arterial fibrosis
TNFα	Macrophages (adipose, placenta)	24–28th	Higher	Pro-inflammatory Insulin resistance Glut4 downregulation
PAI-1	Artery, placenta, adipose	24–28th	Higher	Inhibition of plasminogen Migration of vascular cells
Fetuin-B	Liver, placenta, fetal tissues	24–28th	Higher	Modulation of the insulin receptor Systemic inflammation
Follistatin	Gonadal, intestine, placenta	26th	Lower	Antagonism of activin-A Reduction of cardiac ischaemia–reperfusion injury

Several proteins released at the 24th–28th week of pregnancy in maternal plasma could be useful to diagnose GDM. Some of them have been related with metabolic and cardiovascular pathologies (https://www.genecards.org/)

Other adipose-released factors could be useful for GDM prediction and detection. The fatty acid-binding protein 4 (FABP4) has been correlated with obesity markers, such as high BMI and fat mass, and regulate lipid and glucose metabolism through fatty acid transport and uptake [131]. The retinol-binding protein 4 (RBP4) is a circulating retinol transporter that has been linked with cardiometabolic markers in inflammatory chronic diseases, including obesity, T2DM, metabolic syndrome, and atherosclerosis [132]. Interestingly, high levels of FABP4 were proposed as a predictive biomarker of GDM from at the 1st and 3rd trimester of gestation [133–135]. Also, an upregulation of plasma RBP4 at the 1st and 2nd trimester was modestly correlated with GDM risk, particularly among women with advanced age and obesity [115, 136] (Table 3A). Finally, fibroblast growth factor-23 (FGF-23) is as a multi-functional cytokine with relevant implications in phosphate and vitamin-D metabolism. It also participates in cardiovascular disturbances, including atherosclerosis and left ventricular hypertrophy [137, 138]. Notably, FGF-23 estimated adverse cardiovascular outcomes in women with T2DM [139], and also, high levels of FGF-23 (and low of adiponectin) diagnosed GDM at the 3rd trimester [140] (Table 4).

ii. Placenta-secreted factors

During GDM, some of the previous adipose-derived factors such as TNFα, visfatin, omentin and FABP4 can be also expressed and discharged from placenta, contributing to their elevated plasma levels [141]. Moreover, placenta can co-secrete other factors with potential roles in GDM pathogenesis [142]. Liver-derived sex hormone binding globulin (SHBG) is expressed in placenta as a regulator of sex steroid hormones. SHBG has been inversely linked with obesity, insulin resistance, metabolic syndrome, and T2DM [143]. Remarkably, low plasma SHBG levels in the 1st trimester of gestation was a truly biomarker for GDM [120, 144, 145] (Table 3A). Nanda et al. also observed a reduction of SHBG in parallel to adiponectin in GDM women at the 11–13th week of pregnancy, in association with BMI > 30 kg/m^2^, previous macrosomia and family history of DM [145, 146]. In this line, an hepatokine promoter of insulin resistance, fetuin-B, was increased at the 3rd trimester of GDM pregnancies, and returned after delivery [147] (Table 4). More interestingly, at the late 1st trimester, a reduction of plasma fetuin-A levels (and elevated hs-CRP) was also observed [121] (Table 3A). Another member of the FGF family, FGF-21, which induces the browning of white adipose tissue and acts as an upstream effector of adiponectin, was also expressed in placenta and increased in GDM women at the 24th week of gestation [148]. Also, afamin, a glycoprotein member of the albumin family expressed in liver and other peripheral tissues (i.e., placenta), may serve as an early (1st trimester) biomarker for pathological glucose and lipid metabolism during pregnancy [149]. In this regard, the decreased levels of ficolin-3 (an activator of the lectin pathway of the complement system expressed in liver and placenta) and the increased ratio of ficolin-3/adiponectin were predictive of GDM at the 16–18th week of gestation [115] (Table 3A). Finally, follistatin, a gonadal regulator of follicular-stimulant hormone and activin-A, with angiogenic, anti-inflammatory and cardioprotective properties, were lowered in the 3rd trimester of GDM pregnancy [150] (Table 4).

In addition, non-coding RNAs such as micro-RNAs (miR) can be released from placenta to maternal circulation as early as the 6th week of gestation. They could be involved in placenta development, insulin signalling and cardiovascular homeostasis [151, 152]. More than 600 placental miR are mainly encoded into three genetic cluster [chromosome 19 microRNA cluster (C19MC), C14MC, and miR-371-3 cluster]. These miR can be secreted by passive (associated to argonaute proteins or apoptotic bodies) or active (packaged into shedding vesicles, exosomes or lipoproteins) mechanisms, and regulate trophoblasts proliferation (i.e., mir-376c, miR-141, miR-155, miR-675), apoptosis (i.e., miR-29b, miR-182), migration and invasion (i.e., mir-376c, miR-195, miR-21, miR-29b), and angiogenesis (miR-16, miR-29b, miR-17/92) [153]. However, placental miR can be unbalanced in complicated pregnancies like GDM. A significant downregulation of miR-29a, miR-132 and miR-222 were observed in plasma at the 16th week of pregnant women who developed GDM (Table 3B) [154]. miR-29a has been linked to fatty acid and glucose metabolism, whereas miR-132 was related with incretin-dependent insulin secretion and enhancement of glucose homeostasis, and miR-222 was involved in insulin resistance and pro-atherogenesis [155–157]. By contrast, other miR involved in insulin secretion and signaling such as miR-16-5p, miR-17-5p, miR-19a/b-3p and miR-20a-5p were upregulated and correlated with GDM from the early 1st–2nd trimester [158, 159]. These miRs have been linked to inflammation, insulin resistance, vascular function and anti-apoptosis [160–163]. Likewise, during the 7th–23rd week of gestation, elevated plasma levels of miR-21-3p were associated with GDM [164, 165] (Table 3B). Interestingly, this miR was linked with preeclampsia and insulin resistance [166, 167].

iii. Urine biomarkers

Maternal urine may be also suitable as a source of predictive and diagnostic markers for GDM. The urine metabolome profile of GDM women in the 3rd trimester of pregnancy identified 14 metabolites related with the steroid hormone biosynthesis and tryptophan metabolism that were significantly elevated [i.e., l-tryptophan, l-urobilinogen, ceramide (d18:0/23:0), 21-deoxycortisol, cucurbitacin-C, aspartame] [168] (Table 5). The upregulation of these pathways could trigger insulin resistance and may respond to oxidative stress and inflammation during GDM. Furthermore, earlier detection (at 12th–26th week of pregnancy) of augmented AHBA, 3-hydroxybutanoic acid (BHBA), valine and alanine levels were observed in urine (and plasma) from GDM mothers [50] (Table 5). Again, these patients also exhibited higher excretion of serotonin and related metabolites like l-tryptophan.

**Table 5:**
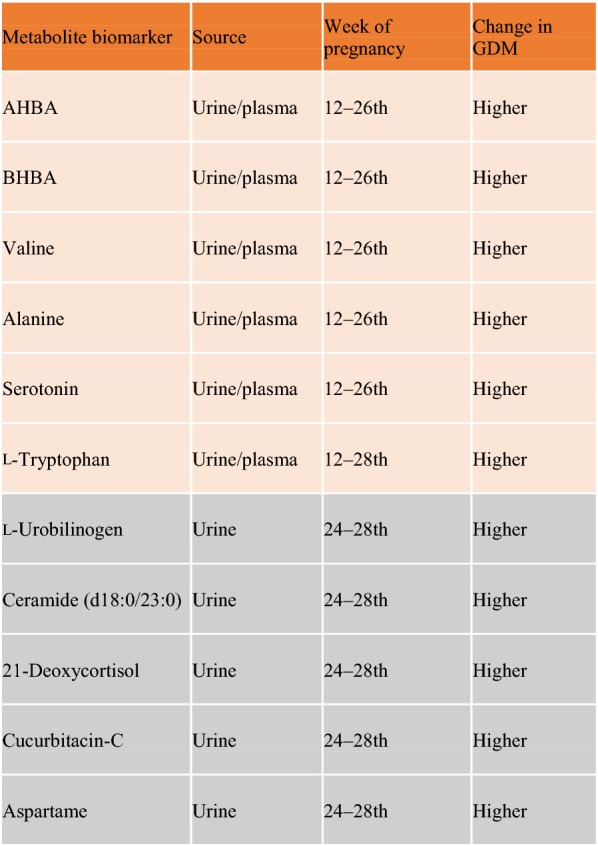
Potential metabolites as biomarkers for GDM

The release of some metabolites at the 12th–28th week of pregnancy to maternal urine or plasma, could be suitable for GDM prediction (in orange) and/or diagnosis (in grey)

## Limitations and future perspectives

An important issue before selecting these biomarkers to clinical practice will be the analysis of their nature and capacity of sensitivity, specificity, accuracy and reproducibility for GDM. Unfortunately, the accuracy and reproducibility cannot be properly described in most cases due to the scarce clinical and validation studies, and to the different origin of patients, timing of sampling and quantitative methodologies. Nevertheless, some protein and miR biomarkers were tested for sensitivity and specificity (Table 6). In particular, SHBG, hs-CRP and FGF-21 reached 85–100% of sensitivity, and miR-16-5p, miR-17-5p and miR-20a-5p attained more than 95% of specificity. Other biomarkers such as leptin, RBP4 and ficolin-3, which displayed 51–81% of sensitivity and over 64% of specificity, improved these parameters when they were related to adiponectin (not shown). Especially, the ficolin-3/adiponectin ratio reached 90.9% and 96.5% of sensitivity and specificity, respectively, for GDM prediction [115]. The sensitivity could have been limited by the variability and quality of samples and detection method, and specificity could have decreased since most biomarkers point out common diabesogenic processes (i.e., insulin resistance, inflammation). In this regard, FGF-21 [148, 169], visfatin [127, 170], IL-6 [119, 171] and resistin [128, 129] displayed variable levels depending on pregnancy phase, and afamin, among others, could serve also as a valid biomarker for other complicated pregnancies, like those with preeclampsia [149]. In this line, GDM patients with cardiovascular risk could be classified by testing biomarkers with key roles on cardiovascular pathophysiology. Altered levels of RBP4, adiponectin, visfatin, fetuin-A, omentin-1, IL-6, FGF-21/23, PAI-1 or several miRs (miR-16-5p, miR-17-5p, miR-20a-5p, miR-222 and miR19a/b-3p) could be suspected for future cardiovascular disorders after GDM (Tables 3A, B and 4). However, no data have evidenced this hypothesis yet. In addition, some biomarkers could show a prognostic role for GDM. The increased concentration of RBP4 in early stages of GDM, was attenuated after sitagliptin treatment and in correlation with insulin resistance [96].

**Table 6:** Sensitivity and specificity of candidate biomarkers for GDM

Biomarker	Week of pregnancy	Sensitivity (%)	Specificity (%)	References
SHGB	1st–12th	85.0	55.3	[121, 145]
hs-CRP	4–6th	89.0	55.3	[121]
	11–14th	86.2	50.8	[122]
FGF-21	24–28th	100.0	75.0	[148]
miR-16-5p	4–6th	41.6	95.8	[159]
miR-17-5p	4–6th	21.4	95.4	
miR-20a-5p	4–6th	17.8	95.4	
FABP4	4–6th	81.8	71.2	[133]
	23rd–30th	87.0	89.0	[135]
Adiponectin	16–18th	80.7	65.1	[117]
	24–28th	83.6	56.6	[113]
RBP4	16–18th	79.4	79.1	[136]
		63.6	75.0	[117]
Leptin	24–28th	81.2	64.2	[113]
miR-132	16th	66.7	63.3	[154]
miR-29a				
miR-222				
Ficolin-3	16–18th	51.1	97.7	[117]
Fetuin-A	11–14th	58.6	76.2	[122]
miR-21-3p	30–36th	52.6	89.3	[165]

Some of the predictive or diagnostic biomarkers (protein and miR) for GDM were analysed for sensitivity and specificity unveiling different data. These parameters, together with reproducibility and accuracy in quantification will be crucial to validate biomarkers for clinical practise

Finally, protein and metabolite biomarkers would deliver a direct measurement of biological effectors involved in GDM, whereas evaluation of miRs could inform about its regulatory mechanisms. Also, modification of protein and miR levels may provide information about specific responses but not about the complete disease. Metabolites, however, usually represent the endpoint or convergence of molecular cascades and is the closest domain to the phenotype, but they exhibit very low reproducibility in validation tests. Thus, integration of data from different molecules could reinforce the understanding and classification of GDM patients by highlighting common pathways that are dysregulated in subsets of patients.

## Conclusion

Nowadays, there is a lack of consensus tools for GDM prediction and diagnosis, which influences on metabolic and cardiovascular evolution for both mother and offspring. After considering risk factors such as increased age, obesity and familiar GDM, specific biomarkers from different stages of GDM pregnancies could be useful for risk stratification and screening of the disease. A 1st trimester decrease of plasma SHBG and adiponectin, in combination with elevated levels of RBP4, afamin, ficolin-3 and certain miR (miR-16-5p, miR-17-5p and miR-20a-5p) could predict GDM with certain warranties (Fig. 1). Quantification of circulant 1,5-anhydroglucitol may also anticipate the GDM development, and later, at the 3rd trimester, a raise of plasma FGF-21 and FABP4 could help an OGTT for GDM diagnosis. In addition, cardiovascular injuries associated to GDM may be predicted or diagnosed by addition of visfatin, omentin-1, fetuin-A, IL-6, PAI-1 and FGF-21/23 to the GDM panel of biomarkers. More research on urine and plasma metabolites (i.e., AHBA, L-tryp) could also propose valid candidates. Prediction and classification of GDM with/without cardiovascular risk would provide an avenue for personalised medicines, addressed specifically targets the main players leading to disease recurrence, and resulting in better clinical outcomes and improvements in quality of life.

## Data Availability

Not applicable.

## Abbreviations

GDM: gestational diabetes mellitus
BMI: body mass index
OGTT: oral glucose tolerance tests
HbA1c: high levels of glycosylated haemoglobin
GCT: glucose challenge test
PRL-GH: prolactin and growth hormone
PAI-1: plasminogen activator inhibitor 1
AHBA: α-hydroxybutyrate
BHBA: 3-hydroxybutanoic acid
IL: interleukin
PTX-3: pentraxin-related gene
CRP: C-reactive protein
SDMA: symmetric dimethylarginine
ADAM: disintegrin and metalloproteinase
TIMP-1: tissue inhibitor of metalloproteinase-1
IGF-1: insulin-like growth factor 1
FABP4: fatty acid-binding protein 4
RBP4: retinol-binding protein 4
FGF-23: fibroblast growth factor-23
SHBG: sex hormone binding globulin
US CARDIA: US Coronary Artery Risk Development in Young Adults
IADPSG: according to the International Association of Diabetes and Pregnancy Study Groups
ADA: American Diabetes Association
NICE: National Institute for Health and Clinical Excellence
ACOG: American College of Obstetricians and Gynaecologists
DGGG: German Association for Gynaecology and Obstetrics
JOGC: Journal of Obstetrics and Gynaecology Canada
NIH: National Institutes of Health
FIGO: International Federation of Gynaecology and Obstetrics
